# The Medical Profession and Politics

**Published:** 1854-07

**Authors:** 


### THE MEDICAL PROFESSION AND POLITICS.

Among the many impressive things addressed by the venerable Chancellor of the University of Maryland to the graduates in his valedictory, when we were taking leave of our *alma mater*, we remember a remark, that physicians should avoid politics. As a general rule, we are convinced by many years of experience since, that the counsel was judicious. Politics are unfavorable to that devotion of the mind to the main pursuit, which is important to the success of the physician. But there are occasions when it is the duty of medical men, in common with all good citizens, to make their opinions and their influence felt. There are great questions upon which it is proper that their voices should be heard. When our liberties or Constitution are in danger, they ought to speak out. But in the politics of physicians there should be nothing sectarian or sectional. Their mental training and social position ought to raise them above fanaticism, and make them generous and catholic in their sympathies and views. With them, in our country, there should be "no South, no North," but our whole, great country. When there arise strife and sectional violence, their aim ought to be to allay it. We rejoice in the existence of a *National* Association which unites the profession of all the States in one body, and we trust that this excellent institution may be perpetual. Its influence, apart from its bearing upon our profession, is most important in a political point of view, and we want all such influences to bind us, as a nation, in closer bonds.

We have a glorious country, with noble institutions. The hope of freedom, all over the world, rests upon us. If we fail, there is no longer hope for free institutions. But our trust is in our Constitution—the supreme law of the land. Unless we preserve that, we can have no assurance of liberty. We must stand by the Constitution. We must respect law.

Here, it seems to us, all physicians, in the North and in the South, can meet and harmonize—in a pledge to abide by the Constitution. No doubt there are questions arising calculated to engender sectional jealousies, and matter enough might be found for angry contentions, but what is to be gained by them? Nay, what is not to be lost? If the different sections of our country were arrayed against each other, feeling that their interests were antagonistic, the Union would be worth nothing, and could not survive a day. But our interests are not antagonistic, and we must practice forbearance towards usages and institutions we do not like. Our government is a compromise, and we must never forget that it is to be maintained by exercising the spirit in which it was first founded. We would say to our friends at the North, you may see a patch on my coat that you don't like, but don't attempt to pull it off; it is fixed more firmly than you suppose, and there are things about it that you do not comprehend. At most, it is a small matter to quarrel about, and if we should fall to belaboring each other on account of it, we should probably gain nothing and lose a great deal by the contest. Let it alone; there are a great many other noble objects before us calling for our joint energies. Let us unite in promoting these. This patch that offends you is one that we must leave to time.

These remarks have been elicited by a recent transaction in one of our great cities with which an eminent member of the medical profession was connected. We refer to the case of the fugitive slave in Boston, in which Dr. J. V. C. SMITH, editor of the *Boston Medical and Surgical Journal*, bore an important part as Mayor of the city. Dr. SMITH acted with firmness and with judgment. He sustained the constitution and the laws, and in doing so has entitled himself to the approbation of his countrymen.

